# Supplementary material for: Modeling glioblastoma heterogeneity as a dynamic network of cell states
Source: Mol Syst Biol. 2021 Sep 16;17(9):e10105. doi: 10.15252/msb.202010105 (PMC8444284; doi:10.15252/msb.202010105)
Supplement: Supplementary file 5 — Source Data for Figure 3 [file MSB-17-e10105-s001.zip › Figure3A_sourcedata/GSEA_3065/nosigdb_state2.GseaPreranked.1622718419215/DALMO_BARCODENUMBER_UNPUB.html]

Details for gene set DALMO\_BARCODENUMBER\_UNPUB[GSEA]

|  || Dataset | state2 |
| Phenotype | NoPhenotypeAvailable |
| Upregulated in class | na\_pos |
| GeneSet | DALMO\_BARCODENUMBER\_UNPUB |
| Enrichment Score (ES) | 0.7172006 |
| Normalized Enrichment Score (NES) | 2.5750968 |
| Nominal p-value | 0.0 |
| FDR q-value | 0.0 |
| FWER p-Value | 0.0 |
Table: GSEA Results Summary

  

Fig 1: Enrichment plot: DALMO\_BARCODENUMBER\_UNPUB      
 Profile of the Running ES Score & Positions of GeneSet Members on the Rank Ordered List

  

| PROBE | GENE SYMBOL | GENE\_TITLE | RANK IN GENE LIST | RANK METRIC SCORE | RUNNING ES | CORE ENRICHMENT || 1 | HIST1H4C |  |  | 0 | 1.309 | 0.0255 | Yes |
| 2 | HIST1H1B |  |  | 1 | 0.902 | 0.0431 | Yes |
| 3 | PCLAF |  |  | 2 | 0.852 | 0.0597 | Yes |
| 4 | HMGB2 |  |  | 4 | 0.823 | 0.0757 | Yes |
| 5 | HIST1H1A |  |  | 7 | 0.760 | 0.0903 | Yes |
| 6 | H2AFZ |  |  | 8 | 0.735 | 0.1046 | Yes |
| 7 | UBE2C |  |  | 9 | 0.704 | 0.1183 | Yes |
| 8 | H2AFX |  |  | 10 | 0.675 | 0.1315 | Yes |
| 9 | DHFR |  |  | 11 | 0.644 | 0.1441 | Yes |
| 10 | UBE2T |  |  | 12 | 0.643 | 0.1566 | Yes |
| 11 | TOP2A |  |  | 15 | 0.596 | 0.1680 | Yes |
| 12 | MKI67 |  |  | 17 | 0.590 | 0.1794 | Yes |
| 13 | CDK1 |  |  | 18 | 0.577 | 0.1906 | Yes |
| 14 | MAD2L1 |  |  | 20 | 0.563 | 0.2015 | Yes |
| 15 | RRM2 |  |  | 22 | 0.556 | 0.2123 | Yes |
| 16 | CKS1B |  |  | 23 | 0.554 | 0.2231 | Yes |
| 17 | HMGB1 |  |  | 25 | 0.549 | 0.2337 | Yes |
| 18 | CKS2 |  |  | 28 | 0.532 | 0.2438 | Yes |
| 19 | TPX2 |  |  | 29 | 0.531 | 0.2542 | Yes |
| 20 | ESCO2 |  |  | 30 | 0.530 | 0.2645 | Yes |
| 21 | DEK |  |  | 31 | 0.526 | 0.2748 | Yes |
| 22 | CENPF |  |  | 34 | 0.518 | 0.2847 | Yes |
| 23 | HMGN2 |  |  | 35 | 0.512 | 0.2947 | Yes |
| 24 | SMC2 |  |  | 39 | 0.493 | 0.3039 | Yes |
| 25 | PBK |  |  | 46 | 0.484 | 0.3127 | Yes |
| 26 | NUSAP1 |  |  | 47 | 0.483 | 0.3222 | Yes |
| 27 | ANP32B |  |  | 48 | 0.474 | 0.3314 | Yes |
| 28 | KPNA2 |  |  | 50 | 0.472 | 0.3405 | Yes |
| 29 | CENPM |  |  | 54 | 0.460 | 0.3491 | Yes |
| 30 | TK1 |  |  | 56 | 0.456 | 0.3579 | Yes |
| 31 | SGO1 |  |  | 58 | 0.449 | 0.3666 | Yes |
| 32 | NCAPG |  |  | 60 | 0.445 | 0.3751 | Yes |
| 33 | BIRC5 |  |  | 61 | 0.443 | 0.3838 | Yes |
| 34 | PTTG1 |  |  | 63 | 0.439 | 0.3922 | Yes |
| 35 | CCNB1 |  |  | 68 | 0.420 | 0.4000 | Yes |
| 36 | SGO2 |  |  | 77 | 0.406 | 0.4070 | Yes |
| 37 | PIMREG |  |  | 80 | 0.398 | 0.4146 | Yes |
| 38 | H2AFV |  |  | 82 | 0.397 | 0.4222 | Yes |
| 39 | KIF4A |  |  | 84 | 0.393 | 0.4297 | Yes |
| 40 | LSM5 |  |  | 85 | 0.393 | 0.4374 | Yes |
| 41 | KIF15 |  |  | 86 | 0.392 | 0.4451 | Yes |
| 42 | CENPN |  |  | 87 | 0.390 | 0.4527 | Yes |
| 43 | GTSE1 |  |  | 89 | 0.389 | 0.4601 | Yes |
| 44 | PHF19 |  |  | 93 | 0.380 | 0.4672 | Yes |
| 45 | CDKN3 |  |  | 97 | 0.376 | 0.4742 | Yes |
| 46 | CDCA5 |  |  | 101 | 0.373 | 0.4812 | Yes |
| 47 | SPC25 |  |  | 104 | 0.372 | 0.4882 | Yes |
| 48 | CENPE |  |  | 105 | 0.372 | 0.4955 | Yes |
| 49 | ANLN |  |  | 111 | 0.358 | 0.5019 | Yes |
| 50 | KIF23 |  |  | 117 | 0.351 | 0.5082 | Yes |
| 51 | ASPM |  |  | 122 | 0.342 | 0.5144 | Yes |
| 52 | TUBB4B |  |  | 125 | 0.340 | 0.5209 | Yes |
| 53 | PTGES3 |  |  | 128 | 0.336 | 0.5272 | Yes |
| 54 | KIF11 |  |  | 129 | 0.335 | 0.5337 | Yes |
| 55 | TACC3 |  |  | 130 | 0.334 | 0.5402 | Yes |
| 56 | POC1A |  |  | 134 | 0.332 | 0.5464 | Yes |
| 57 | CCNA2 |  |  | 135 | 0.331 | 0.5528 | Yes |
| 58 | ANP32E |  |  | 136 | 0.330 | 0.5593 | Yes |
| 59 | ARHGAP11A |  |  | 145 | 0.321 | 0.5647 | Yes |
| 60 | CDCA2 |  |  | 150 | 0.309 | 0.5703 | Yes |
| 61 | CDCA3 |  |  | 160 | 0.304 | 0.5752 | Yes |
| 62 | AURKB |  |  | 163 | 0.303 | 0.5809 | Yes |
| 63 | MZT1 |  |  | 174 | 0.295 | 0.5856 | Yes |
| 64 | ACAT2 |  |  | 180 | 0.294 | 0.5908 | Yes |
| 65 | PAICS |  |  | 187 | 0.291 | 0.5958 | Yes |
| 66 | NUF2 |  |  | 188 | 0.287 | 0.6014 | Yes |
| 67 | CCNB2 |  |  | 190 | 0.286 | 0.6069 | Yes |
| 68 | CDC20 |  |  | 193 | 0.284 | 0.6122 | Yes |
| 69 | HMGB3 |  |  | 200 | 0.282 | 0.6171 | Yes |
| 70 | NCAPG2 |  |  | 201 | 0.282 | 0.6226 | Yes |
| 71 | FOXM1 |  |  | 202 | 0.282 | 0.6281 | Yes |
| 72 | HJURP |  |  | 209 | 0.277 | 0.6328 | Yes |
| 73 | SRSF2 |  |  | 213 | 0.275 | 0.6379 | Yes |
| 74 | SNRPF |  |  | 237 | 0.259 | 0.6404 | Yes |
| 75 | CENPA |  |  | 238 | 0.259 | 0.6455 | Yes |
| 76 | KIF2C |  |  | 252 | 0.251 | 0.6490 | Yes |
| 77 | SPC24 |  |  | 256 | 0.248 | 0.6535 | Yes |
| 78 | MIS18BP1 |  |  | 257 | 0.246 | 0.6583 | Yes |
| 79 | CCT5 |  |  | 279 | 0.242 | 0.6607 | Yes |
| 80 | PRR11 |  |  | 285 | 0.238 | 0.6648 | Yes |
| 81 | CIP2A |  |  | 304 | 0.230 | 0.6674 | Yes |
| 82 | DIAPH3 |  |  | 321 | 0.226 | 0.6700 | Yes |
| 83 | CKAP2L |  |  | 327 | 0.224 | 0.6739 | Yes |
| 84 | POLD2 |  |  | 330 | 0.223 | 0.6780 | Yes |
| 85 | RACGAP1 |  |  | 333 | 0.222 | 0.6821 | Yes |
| 86 | BUB1 |  |  | 335 | 0.222 | 0.6863 | Yes |
| 87 | NCAPH |  |  | 336 | 0.222 | 0.6907 | Yes |
| 88 | PLK1 |  |  | 364 | 0.213 | 0.6919 | Yes |
| 89 | DLGAP5 |  |  | 371 | 0.211 | 0.6954 | Yes |
| 90 | CENPW |  |  | 373 | 0.211 | 0.6994 | Yes |
| 91 | HNRNPM |  |  | 412 | 0.198 | 0.6991 | Yes |
| 92 | RPL35 |  |  | 415 | 0.197 | 0.7027 | Yes |
| 93 | TROAP |  |  | 435 | 0.193 | 0.7044 | Yes |
| 94 | FANCD2 |  |  | 447 | 0.191 | 0.7070 | Yes |
| 95 | SPDL1 |  |  | 459 | 0.189 | 0.7095 | Yes |
| 96 | ENY2 |  |  | 466 | 0.187 | 0.7125 | Yes |
| 97 | LDHA |  |  | 506 | 0.179 | 0.7117 | Yes |
| 98 | LRR1 |  |  | 516 | 0.178 | 0.7142 | Yes |
| 99 | HPRT1 |  |  | 573 | 0.168 | 0.7115 | Yes |
| 100 | KIFC1 |  |  | 577 | 0.167 | 0.7144 | Yes |
| 101 | PARPBP |  |  | 594 | 0.164 | 0.7159 | Yes |
| 102 | CCT8 |  |  | 620 | 0.160 | 0.7163 | Yes |
| 103 | XRCC6 |  |  | 641 | 0.158 | 0.7172 | Yes |
| 104 | SKA3 |  |  | 790 | 0.139 | 0.7039 | No |
| 105 | SMS |  |  | 846 | 0.133 | 0.7006 | No |
| 106 | KIF18B |  |  | 879 | 0.130 | 0.6996 | No |
| 107 | PLK4 |  |  | 986 | 0.121 | 0.6905 | No |
| 108 | DAZAP1 |  |  | 1151 | 0.108 | 0.6749 | No |
| 109 | TCEAL7 |  |  | 1504 | 0.086 | 0.6385 | No |
| 110 | C9orf16 |  |  | 1540 | 0.085 | 0.6364 | No |
| 111 | GADD45A |  |  | 1791 | 0.073 | 0.6108 | No |
| 112 | ODC1 |  |  | 1913 | 0.068 | 0.5990 | No |
| 113 | THOC3 |  |  | 1961 | 0.066 | 0.5952 | No |
| 114 | AKIRIN1 |  |  | 1962 | 0.066 | 0.5965 | No |
| 115 | ATP6V1G1 |  |  | 2201 | 0.057 | 0.5719 | No |
| 116 | S100A10 |  |  | 2608 | 0.044 | 0.5289 | No |
| 117 | PLEKHB1 |  |  | 2927 | 0.036 | 0.4952 | No |
| 118 | VGLL4 |  |  | 3708 | 0.020 | 0.4112 | No |
| 119 | PHPT1 |  |  | 4039 | 0.015 | 0.3759 | No |
| 120 | HMGA1 |  |  | 4135 | 0.013 | 0.3658 | No |
| 121 | RALGAPA2 |  |  | 4425 | 0.008 | 0.3348 | No |
| 122 | NLRP1 |  |  | 5033 | -0.000 | 0.2691 | No |
| 123 | BLOC1S2 |  |  | 5323 | -0.005 | 0.2380 | No |
| 124 | YPEL3 |  |  | 5433 | -0.007 | 0.2264 | No |
| 125 | GSTM3 |  |  | 5666 | -0.011 | 0.2015 | No |
| 126 | PIF1 |  |  | 5869 | -0.014 | 0.1799 | No |
| 127 | NUDT14 |  |  | 5934 | -0.015 | 0.1733 | No |
| 128 | IRX1 |  |  | 6371 | -0.023 | 0.1266 | No |
| 129 | CYSTM1 |  |  | 6438 | -0.024 | 0.1200 | No |
| 130 | SNHG8 |  |  | 6874 | -0.033 | 0.0736 | No |
| 131 | IFI27L2 |  |  | 6887 | -0.034 | 0.0729 | No |
| 132 | FAM210B |  |  | 7076 | -0.038 | 0.0534 | No |
| 133 | RPS27L |  |  | 7139 | -0.040 | 0.0474 | No |
| 134 | CHST7 |  |  | 7208 | -0.042 | 0.0409 | No |
| 135 | KIF3C |  |  | 7422 | -0.048 | 0.0188 | No |
| 136 | RAMP1 |  |  | 7435 | -0.048 | 0.0185 | No |
| 137 | PHLDA3 |  |  | 7590 | -0.053 | 0.0028 | No |
| 138 | RRM2B |  |  | 8015 | -0.068 | -0.0417 | No |
| 139 | MAN2B2 |  |  | 8023 | -0.068 | -0.0411 | No |
| 140 | PCDH9 |  |  | 8035 | -0.068 | -0.0410 | No |
| 141 | GAA |  |  | 8060 | -0.069 | -0.0422 | No |
| 142 | ABCA5 |  |  | 8149 | -0.074 | -0.0503 | No |
| 143 | TRIM22 |  |  | 8154 | -0.075 | -0.0492 | No |
| 144 | GDPD2 |  |  | 8201 | -0.077 | -0.0527 | No |
| 145 | IGSF8 |  |  | 8401 | -0.089 | -0.0725 | No |
| 146 | TMEM59L |  |  | 8529 | -0.099 | -0.0843 | No |
| 147 | ST8SIA1 |  |  | 8559 | -0.101 | -0.0855 | No |
| 148 | CHPF |  |  | 8594 | -0.103 | -0.0872 | No |
| 149 | VPS13C |  |  | 8620 | -0.104 | -0.0878 | No |
| 150 | PROS1 |  |  | 8687 | -0.110 | -0.0928 | No |
| 151 | MDM2 |  |  | 8707 | -0.112 | -0.0927 | No |
| 152 | CTSF |  |  | 8795 | -0.122 | -0.0997 | No |
| 153 | PCMT1 |  |  | 8796 | -0.122 | -0.0973 | No |
| 154 | ATP6V0B |  |  | 8815 | -0.124 | -0.0969 | No |
| 155 | PLXNB2 |  |  | 8819 | -0.125 | -0.0947 | No |
| 156 | PCDHB9 |  |  | 8838 | -0.127 | -0.0942 | No |
| 157 | PLTP |  |  | 8849 | -0.129 | -0.0928 | No |
| 158 | TM2D1 |  |  | 8852 | -0.129 | -0.0905 | No |
| 159 | SEZ6L |  |  | 8858 | -0.130 | -0.0885 | No |
| 160 | TP53I3 |  |  | 8860 | -0.131 | -0.0860 | No |
| 161 | IDS |  |  | 8874 | -0.133 | -0.0848 | No |
| 162 | COL18A1 |  |  | 8909 | -0.137 | -0.0858 | No |
| 163 | APLP1 |  |  | 8911 | -0.137 | -0.0833 | No |
| 164 | COL9A3 |  |  | 8952 | -0.142 | -0.0848 | No |
| 165 | PGRMC1 |  |  | 8958 | -0.143 | -0.0826 | No |
| 166 | ITGA7 |  |  | 9011 | -0.150 | -0.0853 | No |
| 167 | HOOK2 |  |  | 9046 | -0.157 | -0.0859 | No |
| 168 | CHD9 |  |  | 9066 | -0.160 | -0.0848 | No |
| 169 | BEX1 |  |  | 9079 | -0.162 | -0.0830 | No |
| 170 | SEZ6L2 |  |  | 9087 | -0.163 | -0.0806 | No |
| 171 | S100A6 |  |  | 9097 | -0.166 | -0.0783 | No |
| 172 | IP6K2 |  |  | 9136 | -0.174 | -0.0790 | No |
| 173 | HTRA1 |  |  | 9178 | -0.184 | -0.0798 | No |
| 174 | GRIA2 |  |  | 9222 | -0.202 | -0.0806 | No |
| 175 | LONRF2 |  |  | 9228 | -0.204 | -0.0771 | No |
| 176 | BTG2 |  |  | 9239 | -0.207 | -0.0742 | No |
| 177 | ZMAT3 |  |  | 9276 | -0.223 | -0.0737 | No |
| 178 | IGFBP7 |  |  | 9295 | -0.229 | -0.0712 | No |
| 179 | CDKN1A |  |  | 9301 | -0.230 | -0.0672 | No |
| 180 | MMP2 |  |  | 9307 | -0.235 | -0.0632 | No |
| 181 | LAMB2 |  |  | 9319 | -0.246 | -0.0596 | No |
| 182 | PCSK1N |  |  | 9321 | -0.247 | -0.0549 | No |
| 183 | GDF15 |  |  | 9322 | -0.247 | -0.0501 | No |
| 184 | GPC1 |  |  | 9326 | -0.250 | -0.0455 | No |
| 185 | LRP1 |  |  | 9338 | -0.259 | -0.0416 | No |
| 186 | ZBTB20 |  |  | 9341 | -0.261 | -0.0368 | No |
| 187 | DDR1 |  |  | 9345 | -0.266 | -0.0319 | No |
| 188 | ITGAV |  |  | 9346 | -0.269 | -0.0267 | No |
| 189 | SPARCL1 |  |  | 9360 | -0.288 | -0.0224 | No |
| 190 | PSAP |  |  | 9363 | -0.294 | -0.0169 | No |
| 191 | DKK3 |  |  | 9411 | -0.414 | -0.0140 | No |
| 192 | NEAT1 |  |  | 9439 | -0.871 | 0.0001 | No |
Table: GSEA details [plain text format]

  

Fig 2: DALMO\_BARCODENUMBER\_UNPUB: Random ES distribution      
 Gene set null distribution of ES for **DALMO\_BARCODENUMBER\_UNPUB**
